# *Euglena gracilis*-derived β-glucan paramylon entrains the peripheral circadian clocks in mice

**DOI:** 10.3389/fnut.2023.1113118

**Published:** 2023-03-27

**Authors:** Conn Ryan, Siyuan Cao, Masataka Sekiguchi, Atsushi Haraguchi, Ako Murata, Ayaka Nakashima, Kengo Suzuki, Shigenobu Shibata

**Affiliations:** ^1^Laboratory of Physiology and Pharmacology, School of Advanced Science and Engineering, Waseda University, Tokyo, Japan; ^2^Euglena Co., Ltd., Tokyo, Japan

**Keywords:** paramylon, clock genes, short-chain fatty acids, peripheral clock, social jetlag, chrononutrition, *Euglena gracilis*

## Abstract

Paramylon, a β-1,3-glucan storage polysaccharide derived from *Euglena gracilis*, has various health benefits, such as anti-obesity effects and modulation of immune function. However, whether paramylon intake affects the circadian clock remains unknown. In this study, we examined the effect of paramylon intake on the circadian clock. The results showed that the paramylon intake regulated peripheral clocks in mice. Furthermore, cecal pH and short-chain fatty acid concentrations after paramylon intake were measured. The correlation between changes in the expression of clock-related genes and alterations in the intestinal environment was confirmed. In addition, peripheral clock entrainment by paramylon intake was not observed in antibiotic-treated mice whose gut microbiota was weakened. These findings suggest that the regulation of the circadian clock by paramylon intake was mediated by changes in gut microbiota. In addition, the entraining effect of paramylon intake was also confirmed in mice bred under conditions mimicking social jetlag, which implies that paramylon intake may contribute to recovery from social jetlag. Thus, the appropriate consumption of paramylon may have a beneficial effect on health from a chrono-nutritional perspective.

## 1. Introduction

Shift work and irregular lifestyle lead to chronic misalignment of the circadian clock, resulting in sleep disorders, obesity, diabetes, and other lifestyle-related diseases (1). Even in the absence of an irregular lifestyle, the circadian clock can also be disrupted by social jetlag (SJL), which is attributed to the time difference between social time and endogenous time created by the circadian clock system. It is defined as the time difference between weekdays and weekends. SJL has become common in modern days (2, 3).

Most living organisms are born with circadian clocks. The circadian clock is driven by clock genes, such as *Period (Per), Cryptochrome (Cry), Brain and muscle arnt-like protein (Bmal*) and *Circadian locomoter output cycles kaput (Clock*) which form a transcription-translation feedback loop that generates a cycle of approximately 24 h (4). Mammals have two main clocks, a central clock and a peripheral clock. The central clock is located in the suprachiasmatic nucleus (SCN) and is mainly synchronized by light stimuli. The peripheral clock is found in peripheral tissues and cells, such as the liver and kidney, and is normally controlled by the central clock. However, external stimuli, such as food, exercise, and stress, as entrainment factors, may also influence the clock (5).

Chrononutrition is the study of the timing and content of food intake (6). Skipping breakfast and eating late at night can result in a shift of the circadian clock to nighttime, increasing the risk of obesity and diabetes (7). Various functional food components have been reported to contribute to the regulation of the circadian clock, for instance, caffeine (8), and flavonoids (9, 10). Functional food components can contribute to the regulation of the circadian clock and are thought to have beneficial effects on health from a chrono-nutritional perspective. *Euglena gracilis* (*Euglena*) is a microalga that contains a wide range of nutrients including vitamins, minerals, amino acids, and fatty acids. It is often used as a food source or dietary supplement because of its combination of plant and animal characteristics (11). Paramylon, a storage polysaccharide, is an insoluble fiber unique to *Euglena* that consists of straight-chained β-1,3-glucans polymerized into a triple-helical shape (12). Similar to other β-1,3-glucans, paramylon has a variety of health benefits, such as anti-obesity effects (13), suppression of atopic dermatitis (14), anti-HIV effect (15), and modulation of immune functions (16–19). Clinical trials have also shown that continuous intake of *Euglena* improves sleep quality (20). As it is involved in the regulation of sleep, it may be involved in clock regulation; however, no reports have been published examining whether *Euglena* or paramylon is involved in the regulation of the circadian clock. Therefore, research has been conducted focusing on the food components *Euglena* and paramylon, which show various beneficial effects; however, their effects remain unknown from the perspective of chrononutrition. Clock regulation may be associated with the production of short-chain fatty acids (SCFAs), including butyric acid, by gut bacteria (21), The consumption of *Euglena* has been shown to increase the occupancy of *Faecalibacterium* in the gut microbiota and increase butyrate production (22). Hence, we examined how the expression of clock genes and the intestinal environment of mice were altered by feeding them *Euglena*/paramylon mixed in their diets. Additionally, the effects of orally administered *Euglena*/paramylon on PER2:LUC rhythms were examined in the peripheral tissues of mice. To explore the mechanism and applicability in humans, we conducted similar experiments under several conditions.

## 2. Materials and methods

### 2.1. Animals

Male ICR mice (6∼8 weeks old) and male heterozygous PER2:LUC knock-in mice with ICR backgrounds (8∼12 weeks old) were used in this study. ICR mice were purchased from Tokyo Laboratory Animals (Tokyo, Japan) and heterozygous PER2:LUC knock-in mice were bred at Waseda University and housed in the vivarium as previously described (23). ICR mice were used for Experiment 1 while the PER2:LUC knock-in mice were used for Experiment 2 and 3. The mice were maintained under a 12 h light/12 h dark cycle. The lights-on duration was defined as zeitgeber time 0 (ZT0), and the lights-off duration as ZT12. Each mouse was housed in a plastic cage individually at a temperature of 22 ± 2? and humidity of 60 ± 5%. All experimental procedures conformed to the “Fundamental Guidelines for Proper Conduct of Animal Experiment and Related Activities in Academic Research Institutions” (published by the Ministry of Education, Culture, Sports, Science and Technology, Tokyo, Japan) and were approved by the Committee for Animal Experimentation of the School of Science and Engineering at Waseda University (2020-A126, 2021-A051).

### 2.2. Diets and drugs

*Euglena* and paramylon (the nutritional composition of *Euglena* and paramylon is described in Supplementary Table 1 and approximately 70–80% of the carbohydrates in *Euglena* was paramylon) was obtained from Euglena Co., Ltd. (Tokyo, Japan). Paramylon was isolated as previously described (24). Mice were provided with *ad libitum* access to a standard AIN-93M diet (Oriental Yeast Co., Ltd., Tokyo, Japan) and water before the experiment. During the experiment, AIN-93M containing 5% cellulose was used as a control diet, AIN-93M containing 5% *Euglena* (Euglena Co. Ltd., Tokyo, Japan) instead of cellulose was used as a *Euglena* diet, and AIN-93M containing 5% paramylon (Euglena Co. Ltd., Tokyo, Japan) instead of cellulose was used as a paramylon diet (described in Supplementary Table 2). To observe the effects of *Euglena* and paramylon on peripheral clocks by *in vivo* bioluminescence imaging, oral administration was selected instead of feeding experimental diets. *Euglena* and paramylon were administered by adding *Euglena* (50 mg)/paramylon (50 mg) to the vehicle (10 mg/kg body weight).

### 2.3. Antibiotic water treatment

Antibiotic water was produced by mixing 1 g/L of metronidazole (Fujifilm Wako Pure Chemical Co., Osaka, Japan), 1 g/L ampicillin sodium (Fujifilm Wako Pure Chemical Co.), 1 g/L neomycin sulfate (Fujifilm Wako Pure Chemical Co.), and 0.5 g/L of vancomycin hydrochloride (Fujifilm Wako Pure Chemical Co.) in drinking water. While the antibiotic water treatment, mice had access to water bottle filled with antibiotic water *ad libitum*, and no access to tap water. To weaken intestinal flora, mice had been treated antibiotic water for at least 2 weeks.

### 2.4. Cecal pH measurement

Cecal pH was measured using a pH meter (Eutech Instruments, Vernon Hills, IL, USA). A pH meter electrode was inserted directly into the cecum immediately after the cecum sample was collected.

### 2.5. Measurement of SCFAs

Short-chain fatty acids (SCFAs) in cecal contents were measured in cecum samples collected using gas chromatography (7890 B, 5977 B; Agilent Technologies, Inc., Santa Clara, CA, USA). SCFAs were extracted from 50 mg of cecal contents by acidifying the cecal contents with sulfuric acid (Fujifilm Wako Pure Chemical Co., Osaka, Japan) and shaking them in 50 μL of 2 N sulfuric acid, 400 μL of diethyl ether (Fujifilm Wako Pure Chemical Co., Osaka, Japan), and 200 μL of ethanol (Fujifilm Wako Pure Chemical Co., Osaka, Japan). The mixture was centrifuged at 14,000 rpm for 30 s at room temperature. Next, 300 μL of the supernatant was mixed with 100 μL of TMSI-H (GL Science Inc., Tokyo, Japan), and the mixture was heated at 60? for 30 min. After heating, the mixture was placed on ice for 10 min and centrifuged at 14,000 rpm for 30 s at room temperature. A total of 2 μL of the organic phase was injected into a capillary column [InertCap Pure WAX (30 m × 0.25 mm, df = 0.5 μm), GL Science, Tokyo, Japan], followed by analysis by gas chromatography with a flame ionization detector. The incipient and final temperatures were 80? and 200?, respectively. Helium was used as the carrier gas. We have previously established a quantitative analysis of SCFAs from mouse cecal contents (25).

### 2.6. RT-PCR

RNA was extracted from the liver and jejunum using phenol (Omega Bio-Tek, Norcross, GA, USA). Real-time reverse transcription PCR was performed using a One-Step SYBR RT-PCR Kit (Takara Bio Inc., Shiga, Japan) with specific primer pairs (listed in Supplementary Table 3) on a Piko Real PCR system (Thermo Fisher Scientific, Waltham, MA, USA). Primers were designed using the Primer 3 software. The relative expression levels of the target genes were normalized to *Gapdh* expression levels. The data were analyzed using the ΔΔCt method. A melt curve analysis of each primer was performed to identify non-specific products.

### 2.7. *In vivo* bioluminescence imaging

*In vivo* imaging system (IVIS) (Caliper Life Sciences, Hopkinton, MA, USA) was used to monitor the PER2:LUC activity rhythm waveform in the peripheral tissues (the kidneys, liver, and submandibular glands) of the mice. An analytical method to monitor bioluminescence oscillations in peripheral tissues have been developed previously (23). Briefly, the mice were anesthetized with isoflurane (Mylan Inc., Tokyo, Japan) and enriched with oxygen. Mice were anesthetized and injected with d-luciferin potassium salt subcutaneously on the back and near the neck (Promega, Madison, WI, USA) at a dose of 15 mg/kg body weight. Images were captured using an IVIS in the dorsal-up position for the kidney 8 min after the injection and in the ventral-up position for the liver and the submandibular gland 10 min after the injection. Images were obtained six times/day at 4 h intervals (ZT 3, 7, 11, 15, 19, and 23). The mice were returned to their home cages after each imaging procedure. The mice quickly recovered from isoflurane anesthesia. Bioluminescence emitted from the kidneys and liver was calculated automatically using Living Image 3.2 software (Caliper Life Sciences, Perkin Elmer, Waltham, MA, USA). The average photon/min value observed at the six time points on each day was designated as 100%, and the bioluminescence rhythm for the entire day was expressed as a percentage of each set of six time points for the individual organs. The peak phase and amplitude of the normalized data were determined using a single cosinor procedure program (Acro.exe version 3.5). In a previous study, these procedures did not affect peripheral clocks. We established a non-invasive method to measure the oscillation of clock gene expression in peripheral tissues (23).

### 2.8. SJL mimicking conditions

**FIGURE 1:**
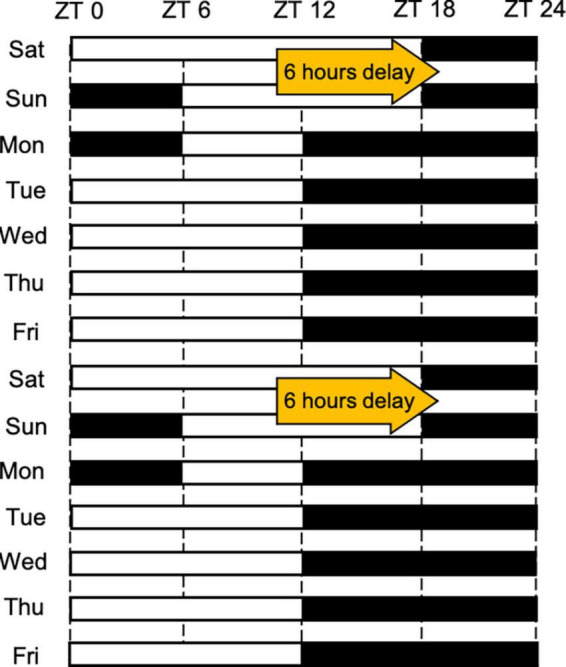
The light schedule followed under conditions mimicking SJL. The white and black bars reflect the on and off status of the light, respectively.

In Experiment 3, all mice were maintained under conditions mimicking SJL for more than 2 weeks before the start of the experiment. Under conditions mimicking SJL, the time of light going on and off was delayed on weekends for 6 h, and thereafter from Monday, normal LD conditions were maintained (light: dark = 12:12) (Figure 1). We established and used the SJL mice model in previous studies (26, 27), and, in this study, we aimed to evaluate the way to reduce the SJL using this SJL mimicking model.

### 2.9. Experimental schedule

**FIGURE 2:**
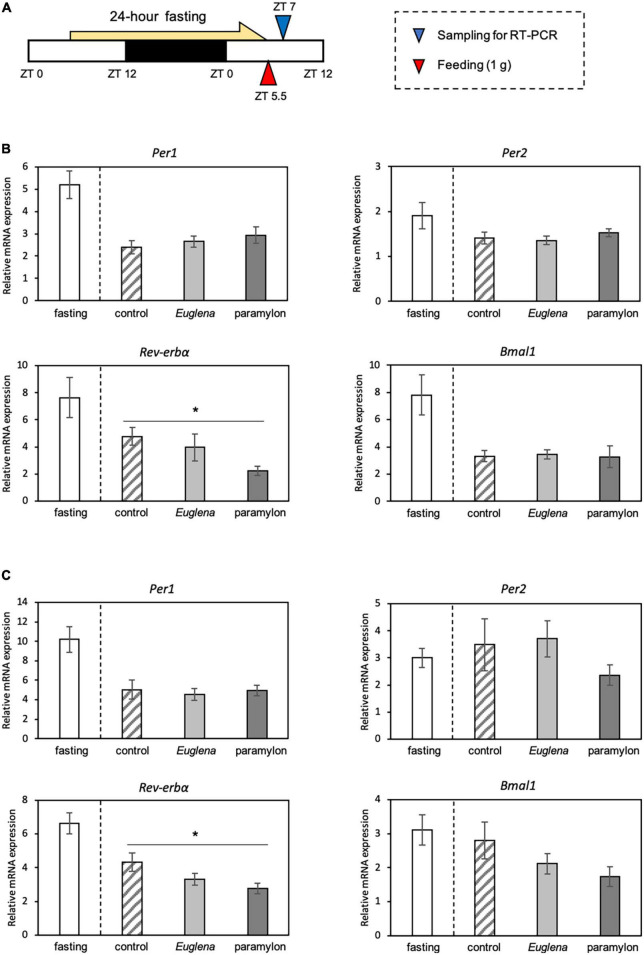
The effects of *Euglena* and paramylon on the expression of genes related to the clock. **(A)** Experimental schedule. The blue arrowhead indicates the sampling time, and the red arrowhead indicates the feeding time. **(B)** Real-time RT-PCR analysis of *Per1, Per2, Rev-erba*, and *Bmal1* mRNA expression in the liver of mice. **(C)** Real-time RT-PCR analysis of *Per1, Per2, Rev-erba*, and *Bmal1* mRNA expression in the jejunum of mice. Values are expressed as mean ± SEM (*n* = 8). * *p* < 0.05, vs. control group (one-way ANOVA with Tukey’s multiple comparisons test).

Experiment 1: All mice experienced 24 h of fasting (ZT5.5 to ZT5.5 of the day after) and had been fed 1 g of experimental diets soon after the fasting. The mice were divided into four groups based on the experimental diet: the fasting group does not have an experimental diet and kept under the fasting condition, the control group was administered the control diet, the *Euglena* group was administered the *Euglena* diet, and the paramylon group was administered the paramylon diet. The mice were euthanized after refeeding, and samples (the liver, jejunum, and cecum) were collected at ZT 7 (Figure 2A).

Experiment 2: Mice were divided into three groups (control, *Euglena*, and paramylon groups) and reagents (vehicle, *Euglena* 50 mg, and paramylon 50 mg) were administered by oral administration (0.1 ml/10 g BW) for three consecutive days at ZT5. As dietary entrainment has a strong effect on the peripheral clock, oral administration was selected instead of experimental diet feeding to adequately examine the effects of the components. 4 h after the final administration, bioluminescence rhythms in the kidney, liver, and submandibular gland were analyzed using *in vivo* imaging system (Figure 4A). The same experimental schedule was conducted for the normal and antibiotic-treated mice.

Experiment 3: The experiment was initiated after the mice were maintained under SJL-mimicking conditions for at least 2 weeks. After experiencing the SJL-mimicking condition, mice (the control group and the paramylon group) were administrated reagents (vehicle or paramylon 50 mg) orally at ZT 12 on Monday, Tuesday and Wednesday. 5 h after the final administration, bioluminescence rhythms in the kidney, liver, and submandibular gland were analyzed using *in vivo* imaging system (Figure 6A).

### 2.10. Statistical analysis

All data are expressed as mean ± standard error of the mean (SEM) and were statistically analyzed using GraphPad Prism version 6.03 (GraphPad Software, San Diego, CA, USA). We determined whether the data showed normal or non-normal distribution and equal or biased variation using the D’Agostino-Pearson test/Kolmogorov-Smirnov test and the *F*-value test/Bartlett’s test, respectively. Parametric analysis was conducted using one-way or two-way ANOVA with a Tukey test, Sidak test, or Student’s *t*-test for *post hoc* analysis. Non-parametric analysis was performed using the Kruskal-Wallis test with Dunn’s test or the Mann-Whitney test for *post hoc* analysis. Correlations among factors were also analyzed using Spearman correlation coefficients. Statistical significance was set at *p* < 0.05.

## 3. Results

### 3.1. Experiment 1: Effects of acute intake of paramylon after fasting on the clock gene expression and intestinal environment in mice

The relationship between Euglena/paramylon and the biological clock has yet to be elucidated. To understand this relationship, we examined whether feeding Euglena/paramylon to mice would alter the expression levels of clock genes.

The expression levels of clock genes were measured in the liver and jejunum samples. Although we measured the expression levels of clock genes of the four experimental groups, we conducted statistical analysis and compared gene expression levels among the three experimental groups: control, *Euglena*, and paramylon. In the current experiment, we could confirm that expression levels of *Rev-erb*α and *Per1* genes were clearly inhibited by fasting conditions as our previous paper (28). The gene expression level of *Rev-erb*α in the liver (*p* < 0.05, Tukey analysis) and jejunum (*p* < 0.05, Tukey’s test) of the paramylon group was significantly lower than that observed in the control (Figures 2B, C). However, other genes, such as *Per1*, *Per2* and *Bmal1* showed similar levels in both *Euglena* and paramylon groups (Figures 2B, C).

**FIGURE 3:**
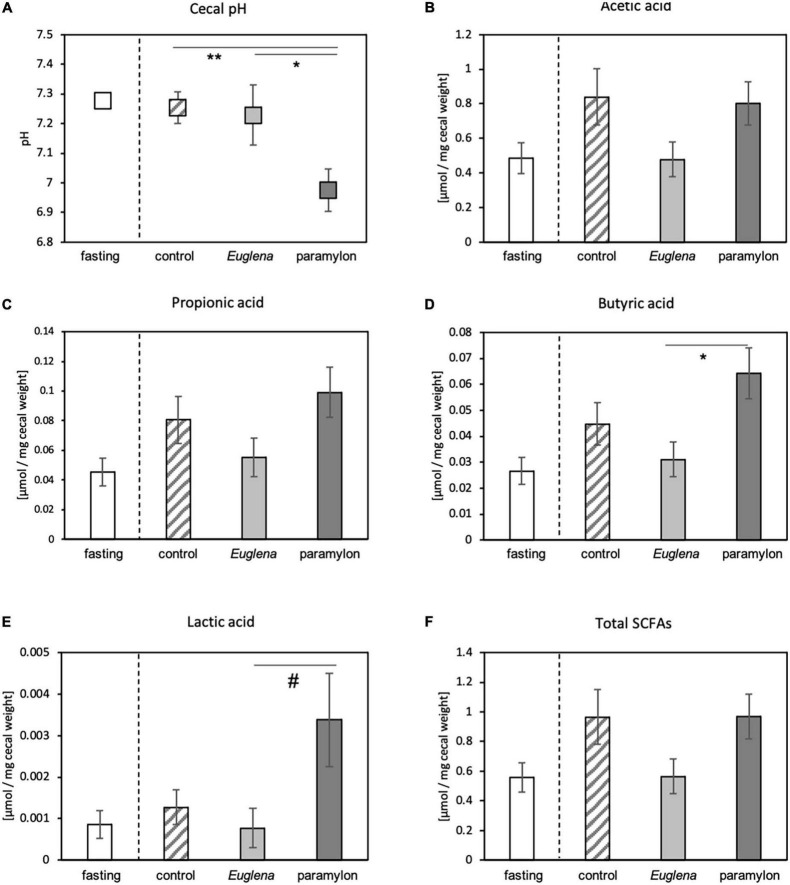
Effects of paramylon diet on cecal pH and the concentration of butyric acid in mice. **(A)** Cecal pH. **(B–F)** Concentrations of SCFAs in the cecal content including **(B)** acetic acid, **(C)** propionic acid, **(D)** butyric acid, and **(E)** lactic acid; **(F)** total SCFA levels. Values are expressed as mean ± SEM (*n* = 8). * *p* < 0.05, ** *p* < 0.01 (one-way ANOVA with Tukey’s multiple comparisons test). ^#^*p* < 0.05 (Kruskal-Wallis test with Dunn’s multiple comparisons test).

Effects of *Euglena*/paramylon consumption on the intestinal environment was also observed by measuring cecal pH and SCFA concentration. Like, the comparison of the expression levels of clock genes, we conducted statistical analysis and compared cecal pH and SCFA concentration among the three experimental groups: control, *Euglena*, and paramylon. The pH of the cecum of the paramylon group was lower than that of the control and *Euglena* group (Figure 3A). The concentrations of acetic acid, propionic acid, butyric acid, and lactic acid were measured in the cecal contents, and we defined the sum of measured SCFA concentrations as total SCFAs (Figures 3B-F). The butyric acid concentration in the paramylon group was significantly higher than that in the *Euglena* group (*p* < 0.05, Tukey’s analysis) (Figure 3D) and the lactic acid concentration in the paramylon group was significantly higher than that in the *Euglena* group (*p* < 0.05, Dunn’s analysis) (Figure 3E). However, no differences were found between groups with respect to acetic acid, propionic acid, or total SCFA levels (Figures 3B, C, and F).

**FIGURE 4:**
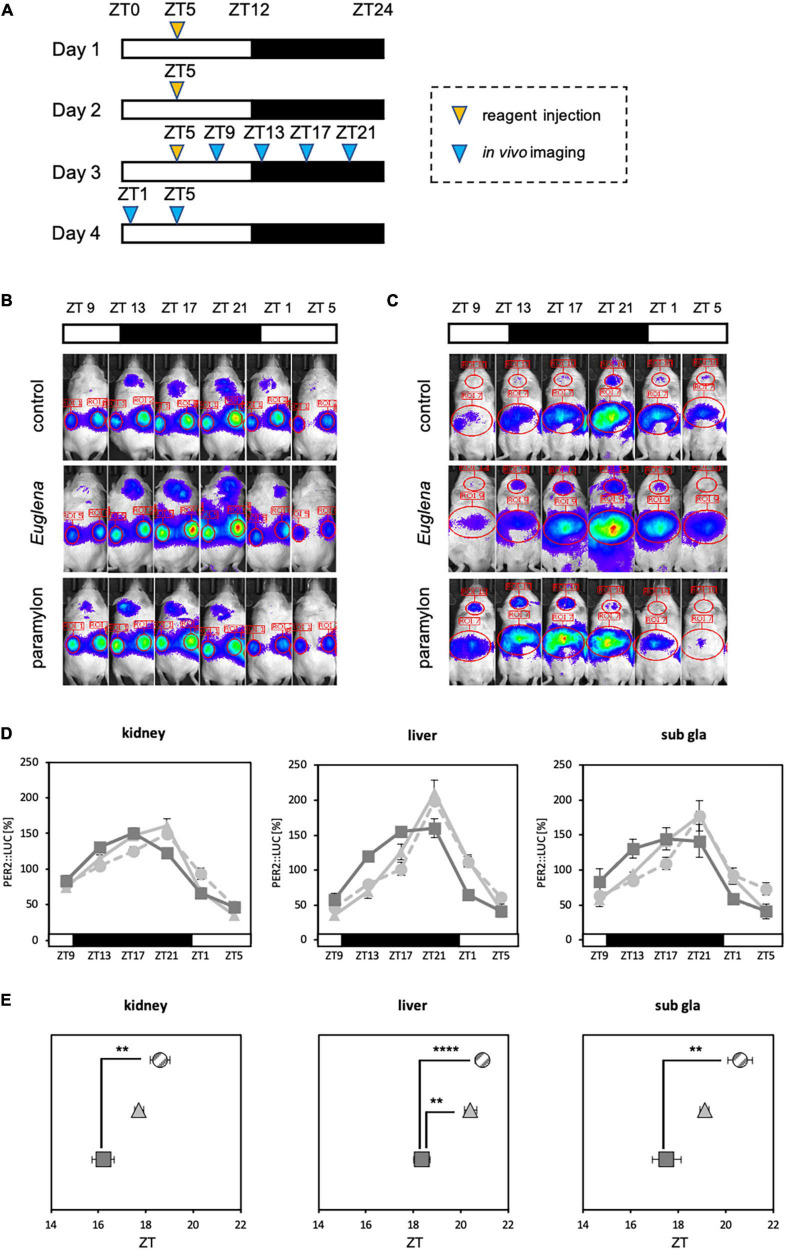
Paramylon intake advances the phase of peripheral clocks. **(A)** Experimental schedule. The yellow arrowhead indicates the reagent administration time, and the blue arrowheads indicate the timings of *in vivo* bioluminescence imaging. **(B,C)** Representative images of *in vivo* bioluminescence imaging. **(B)** The luminescence observed in the kidney. **(C)** The luminescence observed in the liver and submandibular gland. **(D)** Waveforms of PER2:LUC rhythm in each tissue. Light gray dotted lines indicate the control group, light gray lines indicate the *Euglena* group, and dark gray lines indicate the paramylon group. **(E)** Peak phases of PER2:LUC rhythm in each tissue. Gray striped dots indicate the control group, light gray dots indicate the *Euglena* group, and dark gray dots indicate the paramylon group. Values are expressed as mean ± SEM. (control and paramylon group: *n* = 8, *Euglena* group: *n* = 4). ** *p* < 0.01, **** *p* < 0.0001 (one-way ANOVA with Tukey’s multiple comparisons test).

According to these results, paramylon intake appears to affect clock genes and the intestinal environment. Finally, we evaluated the relationship between clock gene expression and SCFA concentrations in the paramylon group to examine whether alterations in clock gene expression are related to alterations in the intestinal environment. *Per2* expression in the liver was significantly positively correlated with acetic acid, butyric acid, and total SCFAs. *Rev-erb*α expression in the liver and jejunum was significantly and positively correlated with cecal pH (Table 1). These results suggest that paramylon affects SCFA and entrains the peripheral clocks.

**TABLE 1:** Correlation between the expression of genes related to the clock and cecal pH/SCFAs of the paramylon group.

		Cecal pH		Acetic acid		Propionic acid		Butyric acid		Lactic acid		Total SCFA	
		***r***	***P***	***r***	***p***	***r***	***P***	***r***	***p***	***r***	***p***	***R***	***p***
liver	*Per1*	−0.108	0.556	−0.164	0.37	−0.182	0.318	−0.185	0.312	0.021	0.909	−0.159	0.385
	*Per2*	−0.122	0.505	0.356	0.046*	0.282	0.118	0.369	0.038*	0.126	0.491	0.354	0.047*
	*Bmal1*	−0.237	0.191	0.069	0.706	0.118	0.519	0.081	0.661	0.078	0.67	0.086	0.638
	*Rev-erb*α	0.383	0.03*	−0.234	0.198	−0.192	0.292	−0.321	0.073	−0.004	0.981	−0.276	0.126
jejunum	*Per1*	0.197	0.287	−0.08	0.669	−0.128	0.492	−0.147	0.429	−0.162	0.384	−0.097	0.605
	*Per2*	0.179	0.336	0.032	0.866	−0.091	0.628	−0.087	0.641	−0.222	0.231	0.015	0.935
	*Bmal1*	0.261	0.157	0.346	0.057	0.268	0.145	0.143	0.444	0.08	0.67	0.311	0.089
	*Rev-erb*α	0.418	0.019*	0.183	0.324	0.057	0.759	−0.013	0.944	−0.22	0.235	0.133	0.475

The colored cells marked with * indicate a significant correlation.

* *p* < 0.05 (Spearman’s correlation coefficient).

### 3.2. Experiment 2: Effects of paramylon administration on peripheral clocks in normal and intestinal flora weakened mice

**FIGURE 5:**
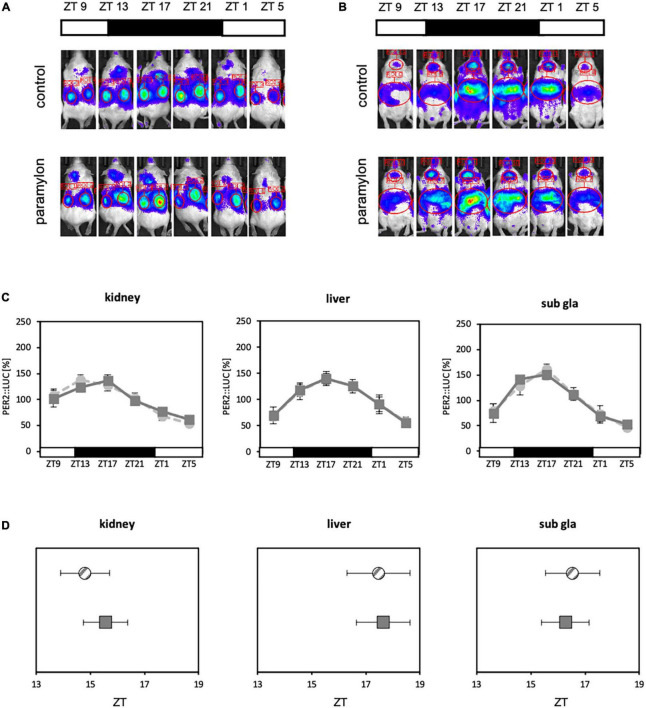
Antibiotic water treatment negates the phase advancing effect of paramylon intake. **(A,B)** Representative images of *in vivo* bioluminescence imaging. **(A)** The luminescence observed in the kidney. **(B)** The luminescence observed in the liver and submandibular gland. **(C)** Waveforms of PER2:LUC rhythm in each tissue. Light gray dotted lines indicate the control group, and dark gray lines indicate the paramylon group. **(D)** Peak phases of PER2:LUC rhythm in each tissue. Gray striped dots indicate the control group, and dark gray dots indicate the paramylon group. Values are expressed as mean ± SEM. (*n* = 6). (Student’s *t*-test) n.s.

Refeeding experiments showed that paramylon consumption affects the expression of clock genes and the intestinal environment. Furthermore, some clock genes that were altered by the paramylon diet were found to be positively correlated with alterations in the intestinal environment. SCFA administered *via* oral gavage can facilitate peripheral clock adjustments (21). Therefore, as paramylon intake affects clock gene expression and increases the levels of some SCFAs, paramylon intake may also show a peripheral clock adjustment effect similar to that observed with the SCFA intake. To verify the peripheral clock adjustment effect of paramylon intake, an experiment was conducted using *in vivo* bioluminescence imaging to observe peripheral clocks (Figures 4B, C). The peak times of oscillatory PER2:LUC activity in the kidney and submandibular gland were significantly phase-advanced in the paramylon group compared to those in the control group. In the liver, the peak time of PER2:LUC oscillation was significantly phase-advanced in the paramylon group compared to those in the control and *Euglena* groups (Figures 4D, E). Paramylon administration in mice demonstrated results similar to those observed when SCFA was administered *via* oral gavage (21). In order to determine if significant phase-advance of PER2:LUC oscillation due to paramylon administration is attributed to alterations in the intestinal environment, we subjected antibiotic-treated mice to the same protocol as that in the previous experiment (Figure 4A). Also, only the paramylon group showed the phase-advance effect of the peak time of PER2:LUC oscillation in the peripheral clocks in the former *in vivo* bioluminescence imaging (Figures 4D, E). Ergo, antibiotic-treated mice were divided into two groups (the control group and the paramylon group) and *in vivo* bioluminescence imaging was conducted to observe peripheral clocks (Figures 5A, B). The peak times of oscillatory PER2:LUC activity in each tissue remained unaltered (Figures 5C, D). Thus, the peripheral clock adjustment effect of paramylon intake disappeared with the antibiotic water treatment. These results suggest that paramylon intake can be an option to entrain peripheral clocks and that alterations in the intestinal environment may be the cause of paramylon-induced entrainment.

**FIGURE 6:**
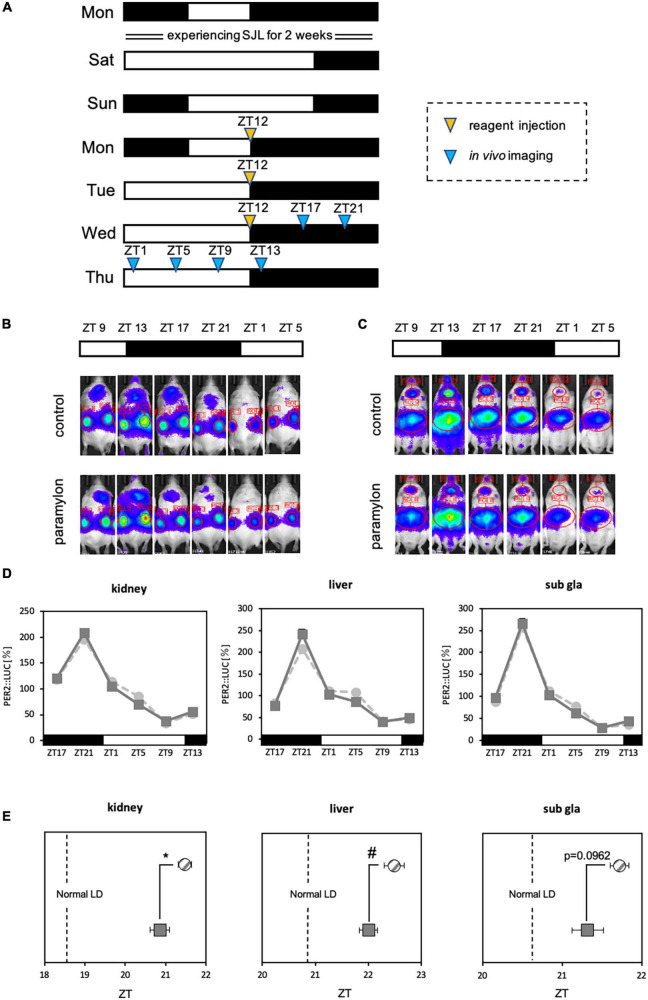
Paramylon intake assists with the reentrainment of peripheral clocks under conditions involving social jetlag. **(A)** Experimental schedule. The yellow arrowhead indicates the reagent administration time, and the blue arrowheads indicate timings of *in vivo* bioluminescence imaging. **(B,C)** Representative images of *in vivo* bioluminescence imaging. **(B)** The luminescence observed in the kidney. **(C)** The luminescence observed in the liver and submandibular gland. **(D)** Waveform of PER2: LUC rhythm in each tissue. Light gray dotted lines indicate the control group, and dark gray lines indicate the paramylon group. **(E)** Peak phase of PER2: LUC rhythm in each tissue. Gray striped dots indicate the control group, and dark gray dots indicate the paramylon group. The vertical dotted lines indicate the phase under the normal LD condition (the control group of Figure 3) (*n* = 10). * *p* < 0.05, vs. control group (Student’s *t*-test), # *p* < 0.05, vs. control group (Mann-Whitney test).

### 3.3. Experiment 3: Effects of paramylon administration on peripheral clocks

The entraining effect of paramylon on peripheral clocks was observed in Experiment 2. Hence, we attempted to apply the entraining effect to SJL, which is the cause of the circadian clock disturbance that has become common in modern days. Experiment 3 was conducted *in vivo* bioluminescence imaging under the SJL-mimicking condition and as the experiment mimicked conditions of human life, the timing of reagent administration was set at the wake-up time of the first 3 days of weekday (Monday, Tuesday, and Wednesday) (Figures 6A-C). Since the lights-off duration is the active phase for mice, the experiment was conducted by administrating paramylon at ZT12, the start of the active phase. The stronger the entraining effect of paramylon, the closer the waveform should be to the rhythm of normal LD with a more regular lifestyle. In the kidney and the liver, the phase of the paramylon group was significantly closer to normal LD than that observed with the control group, while in the submandibular gland, the phase of the paramylon group tended to be closer to normal LD than that observed with the control group (Figures 6D, E). The results suggest that adequate paramylon intake may ease SJL. In the case of this experiment, taking paramylon on Monday, Tuesday, and Wednesday mornings may promote recovery from SJL.

## 4. Discussion

This study has three main findings. First, analysis of clock-related gene expression and oscillatory PER2:LUC activity in peripheral organs revealed that paramylon consumption may be involved in the regulation of chronological time in the peripheral clock of mice. Second, the consumption of paramylon mediated a decrease in cecal pH and an increase in butyrate and lactate production, and a correlation was confirmed between changes in the clock-related gene expression and alterations in the intestinal environment after the consumption of high amounts of paramylon. In addition, peripheral clock entrainment mediated by paramylon intake was not observed in mice whose gut microbiota was weakened by the consumption of antibiotic water, in contrast to that observed in normal mice. These findings suggest that the regulation of the circadian clock by paramylon intake was mediated by changes in the intestinal environment. Finally, the clock alteration effect of paramylon intake was also confirmed in mice bred under conditions mimicking SJL. This suggests that paramylon intake may contribute to recovery from SJL. However, the effect on the biological clock of mice in the *Euglena* group was not as strong as that of the mice in the paramylon group in this study. Approximately 40% of dry weight of *Euglena* we used in this study was paramylon. The difference in the effect on the biological clock between the *Euglena* group and paramylon group may be due to the difference in the total amount of paramylon administrated. In an experiment using a human fecal culture system, butyrate production was confirmed with the addition of *Euglena* but not with the addition of paramylon (22), which is in contrast to our findings. It is possible that the amount of paramylon used may be different and that the experimental technique and environment may differ with the use of mice and the fecal culture system. Furthermore, significant changes in some intestinal microflora have been confirmed in experiments using a chronic kidney disease rat model when they were fed paramylon (29); hence, it is necessary to continue to examine the effects of paramylon intake on the intestinal microflora.

The PER2:LUC expression rhythms in peripheral organs observed in this study were similar to those reported by Tahara et al. (21). In their study, no significant difference was observed when comparing the peak times of PER2:LUC expression rhythms in the peripheral organs of tap water-fed mice and antibiotic-treated mice. However, the administration of SCFAs to sterile mice at ZT5 for three days advanced the peak time of PER2:LUC expression rhythm in peripheral organs (21). This indicates that increased SCFA levels were involved in the entrainment of peripheral clocks. In contrast, Tahara et al. also measured the PER2:LUC expression rhythm in peripheral organs after the following SCFAs were administered; acetic acid, propionic acid, lactic acid, and butyric acid, respectively. Administration of butyric acid, which was the SCFA whose production increased significantly in our study, at ZT5 for three days did not alter the peak time of the PER2:LUC expression rhythm in their report. Nevertheless, diurnal variations in the amount of cecal SCFAs have been reported (21). Furthermore, the dose-time dependence of the entrainment effect of paramylon, which will be discussed in the future, correlates with the diurnal variation in the amount of SCFAs, confirming that the entrainment effect of paramylon is mediated *via* SCFAs, particularly butyric acid and lactic acid. If paramylon affects the peripheral clock *via* SCFAs, one possible pathway is mediated by GLP-1. SCFAs bind to GPR41 and GPR43 in intestinal L-cells, triggering the secretion of GLP-1 and PYY (30, 31). Hence, paramylon intake mediates the secretion of GLP-1 *via* an increase in SCFA levels. Moreover, paramylon also directly stimulates intestinal epithelial cells (32). Stimulated intestinal epithelial cells produce cytokines and peptide hormones. The β-glucan receptor dectin-1 triggers the secretion of the anti-inflammatory cytokines IL-8 and CCL2 (33), and enteroendocrine cells produce ghrelin, serotonin, CCK, PYY, GLP-1, and GIP (33). Furthermore, dietary intake of DHA/EPA increases insulin levels and enhances dietary entrainment *via* the production of GLP-1 (34). Therefore, paramylon intake could stimulate intestinal epithelial cells and induce the production of GLP-1, which may lead to the enhancement of dietary entrainment, such as that observed with DHA/EPA intake. Thus, paramylon intake may induce GLP-1 expression by increasing SCFA levels and stimulating intestinal epithelial cells. GLP-1 binds to GLP-1 receptors on splenic beta cells, promotes insulin secretion, and synchronizes the clock genes (35). Restricted feeding can reset the peripheral clock rhythm, which is called the food-entrained rhythm (5, 6). After the fasting period, refeeding-induced expression of clock-related genes is the first step in the initiation of a new phase of rhythm. We have previously reported that a transient decrease in *Rev-erb*α gene expression was clearly observed in the livers of mice fasted for 24 h (28). In addition, an acute decrease in *Rev-erb*α gene expression can be mimicked by insulin alone or insulin/glucose administration (28). In the present study, paramylon, but not *Euglena*, significantly decreased the expression of *Rev-erb*α in the liver and jejunum compared to that observed in the control group. These results suggest that the paramylon-induced reduction in the *Rev-erb*α expression may be mediated *via* the following steps: increase in butyric acid and lactic acid increase, induction of GLP-1 production, and insulin secretion. Therefore, it is important to measure serum GLP-1 and insulin in the future.

In a previous study on humans comparing different types of breakfasts and chronotypes, the Japanese breakfast group reported a higher inclination to morning chronotype than the cereal group. Analysis of the nutrients in the diet of each group showed that Japanese food was better in many nutritional aspects, particularly in terms of dietary fiber. This dietary fiber richness is thought to be one of the reasons why the Japanese breakfast group showed a higher inclination toward morning chronotype (36). Our study also found that the morning administration of paramylon, a type of dietary fiber, to mice helped them recover from SJL. Therefore, morning paramylon intake may contribute to lifestyle improvement through the morning shift induced by dietary fiber intake and the entrainment effect of paramylon.

In summary, we found that the administration of paramylon to mice affected the circadian clock. Phase fluctuation of peripheral organs was confirmed, suggesting that paramylon administration can contribute to the synchronization of peripheral organs. The entraining effect of paramylon intake could be applied to the resynchronization of the peripheral clocks of mice with disrupted circadian clocks, such as SJL. In addition, the effect of paramylon intake on peripheral clocks identified in this study may be one of the mechanisms of action for improved sleep quality, as demonstrated by examining *Euglena* in the previous clinical trial (20). Thus, the consumption of paramylon at the right time may have a beneficial effect on health from a chrono-nutritional perspective. However, the effect of paramylon intake timing on the circadian clock has not yet been examined, and it is necessary to conduct experiments to examine the multiple patterns of intake times. Although our results suggest that the entraining effect of paramylon intake on peripheral clocks can be attributed to alterations in the intestinal environment, further research is necessary to understand the mechanism of action.

## Data availability statement

The raw data supporting the conclusions of this article will be made available by the authors, without undue reservation.

## Ethics statement

The animal study was reviewed and approved by all experimental procedures conformed to the “Fundamental Guidelines for Proper Conduct of Animal Experiment and Related Activities in Academic Research Institutions” (published by the Ministry of Education, Culture, Sports, Science and Technology, Japan) and were approved by the Committee for Animal Experimentation of the School of Science and Engineering at Waseda University (2020-A126 and 2021-A051).

## Author contributions

AH, AN, and SS: conceptualization. CR, SC, MS, AH, AM, and AN: methodology and data collection. CR and AH: formal analysis. CR: writing—original draft preparation and visualization. AH, AM, AN, and SS: writing—review and editing. KS and SS: supervision. SS: funding acquisition. All authors have read and agreed to the published version of the manuscript.
