# Supplementary material for: Euglena gracilis-derived β-glucan paramylon entrains the peripheral circadian clocks in mice
Source: Front Nutr. 2023 Mar 27;10:1113118. doi: 10.3389/fnut.2023.1113118 (PMC10084324; doi:10.3389/fnut.2023.1113118)
Supplement: Supplementary file 1 [file Table_1.DOCX]

Supplementary Material

# Supplementary Figures and Tables

**Supplementary TableS1. Nutritional composition of *Euglena* and paramylon**

|  | *Euglena* | paramylon |
| --- | --- | --- |
| Water | 3.7 g | 3.3 g |
| Protein | 32.5 g | <0.1 g |
| Fat | 8.8 g | 0.5 g |
| Ash | 4.0 g | 0.3 g |
| Carbohydrate | 51.0 g | 95.9 g |
| Total | 100 g | 100 g |

**Supplementary TableS2.** **Composition of the experimental diets**

|  | control diet | *Euglena* diet | paramylon diet |
| --- | --- | --- | --- |
|  | % | % | % |
| Cornstarch | 46.5692 | 46.5692 | 46.5692 |
| Casein | 14.0 | 14.0 | 14.0 |
| Dextrinized cornstarch | 15.5 | 15.5 | 15.5 |
| Sucrose | 10.0 | 10.0 | 10.0 |
| Soybean oil | 4.0 | 4.0 | 4.0 |
| Cellulose | 5.0 | - | - |
| *Euglena* | - | 5.0 | - |
| Paramylon | - | - | 5.0 |
| Mineral mix (AIN-93M-MX) | 3.5 | 3.5 | 3.5 |
| Vitamin mix (AIN-93-VX) | 1.0 | 1.0 | 1.0 |
| L-Cystine | 0.18 | 0.18 | 0.18 |
| Choline bitartrate | 0.25 | 0.25 | 0.25 |
| *tert*-Butylhydroquinone | 0.0008 | 0.0008 | 0.0008 |

**Supplementary TableS3. Primer sequences for RT-PCR analyses**

| *Tbp* | CAGCCTCAGTACAGCAATCAAC |
| --- | --- |
|  | TAGGGGTCATAGGAGTCATTGG |
| *Per1* | CAAGTGGCAATGAGTCCAACG |
|  | CGAAGTTTGAGCTCCCGAAGTG |
| *Per2* | CTGCTAATGTCCAGTGAGAG |
|  | GTACAGGATCTTCCCAGAAAC |
| *Rev-erbα* | CTTCCGTGACCTTTCTCAGC |
|  | CAGCTCCTCCTCGGTAAGTG |
| *Bmal1* | CCACCTCAGAGCCATTGATACA |
|  | GAGCAGGTTTAGTTCCACTTTGTCT |
